# Stroke in hereditary hemorrhagic telangiectasia patients. New evidence for repeated screening and early treatment of pulmonary vascular malformations: two case reports

**DOI:** 10.1186/1471-2377-11-84

**Published:** 2011-07-09

**Authors:** Espartaco Ribeiro, Julien Cogez, Emmanuel Babin, Fausto Viader, Gilles Defer

**Affiliations:** 1 Service de Neurologie, Centre Hospitalier Universitaire Côte de Nacre, 14033 cedex 9, Caen, France; 2 Service d'Oto-Rhino-Laryngologie, Centre Hospitalier Universitaire, Caen, France

## Abstract

**Background:**

Paradoxical embolism due to pulmonary arteriovenous malformations is the main mechanism of brain infarction in patients with hereditary hemorrhagic telangiectasia. International Guidelines have recently been published to clarify the performance of screening tests and the effectiveness of treatment for pulmonary arteriovenous malformations.

**Case Presentation:**

We present two cases of hereditary hemorrhagic telangiectasia patients of our hospital who experienced an acute stroke secondary to paradoxical embolism.

**Conclusions:**

These two cases show that the guidelines must be followed to prevent the occurrence of ischemic stroke in patients with hereditary hemorrhagic telangiectasia, and that although they may be adequate in most cases, there are some patients who need a more personalized approach.

## Background

Hereditary hemorrhagic telangiectasia (HHT) is an autosomal dominant disease with variable penetrance and an estimated prevalence of 1/5000 [1]. Strokes are a major complication, occurring in 10 to 19% of HHT patients [2]. Paradoxical embolism due to pulmonary arteriovenous malformations (PAVMs), which are present in approximately 15-50% of patients with HHT, is the main mechanism of brain infarction [3]. Embolization of PAVMs has proven to be a safe and effective treatment in this situation [4]. We present two cases of acute ischemic stroke secondary to paradoxical embolism in HHT patients in order to discuss the performance of screening tests and the effectiveness of treatment for PAVMs published in the HHT International Guidelines in 2009 [5].

## Case Presentations

### Patient 1: a 35-year-old man with a SMAD4 mutation

**Figure 1:**
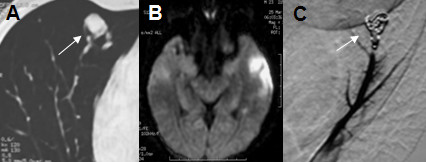
**Radiological features of patient 1**. A: Contrast-enhanced thoracic CT scan shows PAVMs (arrow); B: Brain MRI diffusion hyperintensity in the left temporal cortical region; C: Pulmonary angiography showing PAVMs after embolization (arrow).

In February of 2003, an abnormality was detected on a chest x-ray by an occupational physician. A thoracic CT scan showed asymptomatic PAVMs with a feeding artery diameter (FAD) of 2.4 mm (Figure 1A). One month later, the patient had sudden-onset weakness in the right hand and Wernicke aphasia which spontaneously resolved in 1 hour. The diffusion-weighted MRI showed a left temporal infarct (Figure 1B), and right apex lobe PAVMs were confirmed by pulmonary angiography. Thrombophilia work-up and venous ultrasonography were normal. Other etiologic investigations were negative and the stroke was attributed to paradoxical embolisms. Embolization of the PAVMs was successfully performed (Figure 1C) and antiplatelet therapy was started. No stroke recurrence or complications were observed during 7 years of follow-up.

### Patient 2: a 36-year-old woman with an ENG mutation

**Figure 2:**
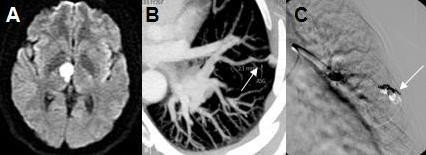
**Radiological features of patient 2**. A: Brain MRI diffusion hyperintensity in the right anterior-medial thalamus region; B: Contrast-enhanced thoracic CT scan shows PAVMs (arrow); C: Pulmonary angiography shows second PAVMs embolized (arrow).

This patient was diagnosed with HHT in 1995 and was regularly followed-up in the Otorhinolaryngology and Pneumology departments. In 2005, she complained of dyspnea and repeated hemoptysis that led to the discovery of a PAVM in the right pulmonary lobe, which was treated by coil embolization. In October 2009, the patient experienced gait disturbance and weakness of the right arm which lasted for 60 minutes. The clinical examination revealed a skew deviation of gaze that disappeared in 48 hours. The transthoracic contrast echocardiography showed an extracardiac shunt and the venous ultrasonography revealed a bilateral deep venous thrombosis (DVT) in both legs. The diffusion-weighted MRI showed right midbrain and anterior-medial thalamic infarctions (Figure 2A). New PAVMs were found in the right lower lobe using a thoracic CT scan (Figure 2B). Other investigations were negative. A paradoxical embolism from the DVT was thought to be the cause of stroke. A coil embolization was successfully performed (Figure 2C) and treatment with warfarin started. No recurrence or complication has been observed in the following 2 years.

## Conclusion

International Guidelines were published in 2009 to clarify the performance of screening tests and the effectiveness of treatment for PAVMs. The selection of PAVMs for embolization is based on feeding artery diameter, generally 3 mm or greater, though targeting PAVMs with a feeding artery diameter as low as 2 mm may be appropriate [5]. Asymptomatic PAVMs were discovered in the first patient with an FAD less than 3 mm. He was not treated in agreement with the international recommendations. Unfortunately a stroke occurred one month later and embolization was finally performed. This confirms that a paradoxical embolism may occur when the FAD is less than 3 mm and embolization of PAVMs should always be discussed.

For patients who have PAVMs, the expert panel recommends that clinicians provide long-term follow-up, consisting of a first screening 6 to 12 months after treatment, then screenings approximately every 3 years after embolization with a multidetector thoracic CT scan, in order to detect growth of untreated PAVMs as well as reperfusion of treated PAVMs. A first screening 6 to 12 months after treatment then approximately every 3 years after embolization with a multidetector thoracic CT scan [5]. The second patient had a past medical history of PAVMs embolized in May 2005. Regular follow-ups with respiratory functional explorations and pulmonary x-rays were performed, but they could not prevent the occurrence of stroke. This case confirms the importance of monitoring patients using the multidetector thoracic CT with thin-cut (eg.1-2 mm) reconstructions and not with other methods that appear less efficient.

Therefore, this work show first, the necessity of closely monitoring patients with HHT in accordance with the international guidelines, and second, that treatment of PAVMs with transcatheter embolotherapy should be discussed on a case by case basis no matter the size of the FAD.

## Consent

Written informed consent was obtained from the patients for publication of these cases reports and any accompanying images. A copy of the written consent is available for review by the Editor-in-Chief of this journal.

## List of abbreviations

PAVMs: Pulmonary arteriovenous malformations; HHT: Hereditary hemorrhagic telangiectasia; MRI: Magnetic resonance imaging; DVT: Deep venous thrombosis; FAD: Feeding artery diameter; CT: Computed tomography.

## Conflict of Interest/Disclosure

The authors declare that they have no competing interests.

## Authors' contributions

ER was responsible for overall study design, management, analysis, and writing.  JC was equally responsible for the design, management, and analysis in the study. EB, FV, and GD provided technical oversight to the study, which included reviewing writing parts of the manuscript.   

All authors read and approved the final manuscript

## Pre-publication history

The pre-publication history for this paper can be accessed here:

http://www.biomedcentral.com/1471-2377/11/84/prepub
